# Clinical evaluation of molecular surrogate subtypes in patients with ipsilateral multifocal primary breast cancer

**DOI:** 10.1186/s13058-023-01632-5

**Published:** 2023-04-06

**Authors:** Slavica Janeva, Ellen Krabbe, Toshima Z. Parris, Salmir Nasic, Marie Sundquist, Per Karlsson, Riccardo A. Audisio, Roger Olofsson Bagge, Anikó Kovács

**Affiliations:** 1 Department of Surgery, Sahlgrenska Breast Center, Sahlgrenska University Hospital, Region Västra Götaland, Gothenburg, Sweden; 2 Department of Clinical Pathology, Institute of Biomedicine, Sahlgrenska Academy, University of Gothenburg, Gothenburg, Sweden; 3 Department of Surgery, Kungälv Hospital, Region Västra Götaland, Kungälv, Sweden; 4 Department of Oncology, Institute of Clinical Sciences, Sahlgrenska Academy, University of Gothenburg, Gothenburg, Sweden; 5 Sahlgrenska Center for Cancer Research, Sahlgrenska Academy, University of Gothenburg, Gothenburg, Sweden; 6 Research and Development Centre, Skaraborg Hospital, Skövde, Sweden; 7 Department of Molecular and Clinical Medicine, Institute of Medicine, Sahlgrenska Academy, University of Gothenburg, Gothenburg, Sweden; 8 Department of Surgery, Kalmar County Hospital, Kalmar, Sweden; 9 Department of Oncology, Sahlgrenska University Hospital, Region Västra Götaland, Gothenburg, Sweden; 10 Department of Surgery, Institute of Clinical Sciences, Sahlgrenska Academy, University of Gothenburg, Gothenburg, Sweden; 11 Wallenberg Centre for Molecular and Translational Medicine, University of Gothenburg, Gothenburg, Sweden; 12 Department of Clinical Pathology, Sahlgrenska University Hospital, Region Västra Götaland, Gothenburg, Sweden

**Keywords:** Multifocal breast cancer, Multicentric breast cancer, Biomarkers, Breast cancer subtypes, Adjuvant treatment, Synchronous, Discordant

## Abstract

**Background:**

When ipsilateral multifocal primary breast cancer (IMBC) is detected, standard routine is to evaluate the largest tumor with immunohistochemistry (IHC). As all foci are not routinely characterized, many patients may not receive optimal adjuvant treatment. Here, we assess the clinical relevance of examining at least two foci present in patients with IMBC.

**Methods:**

Patients diagnosed and treated for IMBC at Sahlgrenska University Hospital (Gothenburg, Sweden) between 2012 and 2017 were screened. In total, 180 patients with ≥ 2 invasive foci (183 specimens) were assessed with IHC and included in this study. Expression of the estrogen (ER) and progesterone (PR) receptors, Ki67, HER2, and tumor grade were used to determine the molecular surrogate subtypes and discordance among the foci was recorded. An additional multidisciplinary team board was then held to re-assess whether treatment recommendations changed due to discordances in molecular surrogate subtype between the different foci.

**Results:**

Discordance in ER, PR, HER2, and Ki67 was found in 2.7%, 19.1%, 7.7%, and 16.9% of invasive foci, respectively. Discordance in the molecular surrogate subtypes was found in 48 of 180 (26.7%) patients, which resulted in therapy changes for 11 patients (6.1%). These patients received additional endocrine therapy (*n* = 2), chemotherapy (*n* = 3), and combined chemotherapy and trastuzumab (*n* = 6).

**Conclusion:**

Taken together, when assessing at least two tumor foci with IHC, regardless of shared morphology or tumor grade between the different foci, 6.1% of patients with IMBC were recommended additional adjuvant treatment. A pathologic assessment using IHC of all foci is therefore recommended to assist in individualized treatment decision making.

**Supplementary Information:**

The online version contains supplementary material available at 10.1186/s13058-023-01632-5.

## Introduction

Multifocal breast cancer has a reported incidence of 4–75% [1, 2] and occurs when two or more synchronous ipsilateral tumors affect the mammary gland. Studies show the lack of consistency regarding not only the incidence rates, but also the definition [1, 3], survival and prognosis [4–8], surgical strategy [9–11], assessment of the different foci [12–15], classification of the size(s) [16–18], and the origin of the multiple tumors found [12, 19, 20]. However, standard of care includes immunohistochemical (IHC) evaluation of only the largest tumor for the expression of the estrogen/progesterone receptors (ER/PR), human epidermal growth factor 2 (HER2), and the proliferation marker Ki-67. The other focus or foci are only further assessed whether the morphology and grade differ from the largest tumor [18, 21]. These tumor characteristics are then used to determine the breast cancer molecular surrogate subtype: luminal A, luminal B HER2-negative, luminal B HER2-positive, non-luminal HER2-positive, triple-negative). While not mandated by consensus guidelines, there are many institutions that do perform IHC assessment on all tumor foci as routine practice on multifocal breast cancer.

Adjuvant treatment recommendations (e.g., endocrine treatment, HER2-targeted treatment, chemotherapy, radiotherapy) depend on patient (age, co-morbidities) and tumor characteristics (size, grade, nodal status, tumor subtype). A number of gene expression assays (e.g., Oncotype Dx, Mammaprint, and Prosigna) have also shown predictive and prognostic capabilities, but these are primarily used to give additional information concerning adjuvant chemotherapy and do not currently replace IHC [22–24]. However, little is known about the clinical relevance of analyzing all foci present with IHC for patients with IMBC [13, 25].

As all foci are not routinely evaluated, we hypothesize that many patients with multifocal breast cancer may not receive optimal adjuvant treatment. The aim of this study was to determine the concordance of histologic type, tumor grade, biological markers (ER, PR, HER2 and Ki67), and molecular surrogate subtypes between the largest tumor and other foci in IMBC where ≥ 2 tumor foci were evaluated with IHC. Furthermore, we investigate the clinical implications of discordance in the choice of adjuvant treatment for these patients.

## Materials and methods

### Study population

In this retrospective study, women diagnosed and treated for primary multifocal breast cancer at Sahlgrenska University Hospital (Gothenburg, Sweden) between 2012 and 2017 were screened for inclusion. Inclusion criteria were specimens with ≥ 2 invasive foci and ≥ 2 tumors with information for the ER, PR, HER2 and Ki67 breast cancer biomarkers assessed with IHC. Exclusion criteria were prior breast cancer-related surgery in the same breast, patients who received neoadjuvant treatment, and distant metastases found at the time of diagnosis. Patient data were retrieved from the Swedish National Breast Cancer Register and local hospital records (i.e., Melior for patient medical records and Sympathy for local pathology reports). Ipsilateral multifocal primary breast cancer was defined as more than one invasive tumor reported in the pathology report regardless of the distance between the foci or multiple invasive tumors found within the same quadrant. The tissue between two invasive foci could consist of normal breast tissue or ductal carcinoma in situ (DCIS). The study was approved by the Regional Ethical Review Board in Linköping, Sweden (registration number 2016/387-31) and the Regional Ethical Review Board in Gothenburg, Sweden (registration number 479--18).

### Immunohistochemistry and receptor-based molecular surrogate subtypes

At the time of clinical evaluation, IHC was performed using 4-µm formalin-fixed, paraffin-embedded sections with the following antibodies: rabbit anti-ERα (Agilent Dako IR084, clone EP1), mouse anti-PR (Agilent Dako IR068, clone 636), mouse anti-KI-67 (Agilent Dako IR626, clone MIB-1), and rabbit anti-human HercepTest (Agilent Dako SK001). In Sweden, (2012–2017), ER and PR were considered positive with ≥ 10% immunostaining in neoplastic cells. Ki67 index was considered to be high with ≥ 14% immunostaining in neoplastic cells between 2012–2013, ≥ 30% immunostaining in neoplastic cells between 2013 and 2016, and ≥ 20% immunostaining in neoplastic cells in 2017. Samples with HercepTest scores of 2 + and 3 + were confirmed for amplification using silver in situ hybridization.

The receptor-based molecular surrogate subtypes (luminal A, luminal B HER2-negative, luminal B HER2-positive, non-luminal HER2-positive, triple-negative breast cancer [TNBC]) were determined for each focus using the applicable Swedish National guidelines for the biomarkers as mentioned above, i.e., luminal A: ER + , PR ± , HER2-, Ki67 low; luminal B HER2-negative: ER + , PR ± , HER2-, Ki67 high; luminal B HER2-positive: ER + , PR ± , HER2 + , any Ki67; non-luminal HER2-positive: ER-, PR-, HER2 + ; TNBC: ER–, PR–, HER2– [26]. Data on co-morbidities and postoperative adjuvant treatment were collected from patient medical records and applicable national treatment guidelines were considered when an additional multidisciplinary team meeting was held to reevaluate the treatment received by patients with discordant subtypes (IHC assessed samples) between the largest primary tumor (PT1) and the second tumor (PT2).

### Statistical analysis

Statistical comparisons were performed only using two foci per specimen. Descriptive statistics as frequencies and percentages were presented for categorical variables and median with quartiles for continuous variables. Concordance between PT1 and PT2 with respect to categorical characteristics was explored by two-way crosstabs. Comparisons between PT1 and PT2 with respect to numerical variables were tested by nonparametric test for related measurements (Wilcoxon’s test), while comparisons between PT1 and PT2 with respect to dichotomous variable were tested by McNemar’s test. Independent groups were compared by Chi-square test or Mann–Whitney test. IBM SPSS v 28 was used for descriptive and analytic statistics.2. Kaplan–Meier plots were constructed for overall survival (OS) and disease-free survival (DFS) using the ggsurvfit package (v0.1.0) in R/Bioconductor (v4.1.1) [27]. OS was defined as the time from primary surgery to death of any cause, and DFS was defined as the time from primary surgery to local recurrence or distant metastasis or death. End of follow-up was 5th of October 2022. The tableone R script (version 0.13.2) was used to identify clinicopathologic features between different groups [28].

## Results

### Patient characteristics

**Fig. 1:**
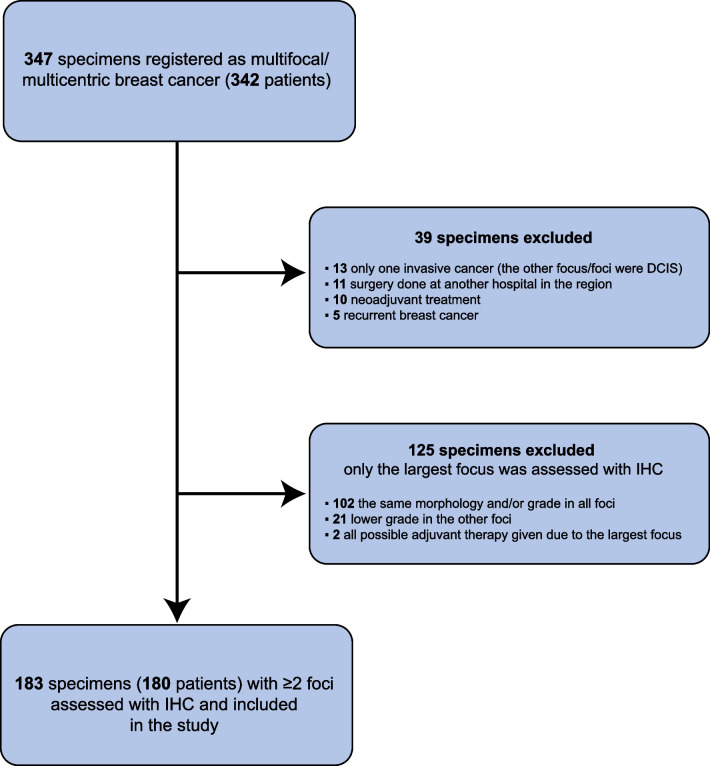
Flowchart of patients with ipsilateral multifocal breast cancer surgically treated at Sahlgrenska University Hospital (Gothenburg, Sweden) during 2012–2017

Between 2012 and 2017, 3362 primary invasive breast cancer specimens obtained by surgical resection were registered at Sahlgrenska University Hospital (Gothenburg, Sweden). Of the 3362 specimens, 347 (10.3%) contained ≥ 2 tumor foci. As only one focus was assessed with IHC, 125 of the 347 breast specimens (34.6%) were excluded. In total, 183 breast specimens from 180 patients (three patients had surgery done for bilateral IMBC) had ≥ 2 foci assessed with IHC and were therefore included in the study (Fig. 1).

Patient characteristics for the 180 patients and 183 specimens are shown in Table 1. Median age was 60 years (IQR 52–71). The most common surgery in the breast was mastectomy (71.6%), and in the axilla sentinel lymph node biopsy was the most common surgical procedure (70.0%). The majority of the patients received adjuvant endocrine therapy (93.3%) and 64 (35.5%) received chemotherapy. Most of the tumors were bifocal (77.0%).

**Table 1:** Characteristics of patients with ipsilateral multifocal primary breast cancer

No. of patients (*n*)	180
No. of specimens (*n*)	183
Age, years (median, IQR)	60 (52–71)
Surgery, breast	
Mastectomy	131 (71.6)
Breast-conserving surgery	52 (28.4)
Surgery, axilla	
SLNB only	128 (70.0)
SLNB + ALND^a^	25 (13.7)
ALND upfront	21 (11.5)
No axillary surgery performed	9 (4.9)
No. of primary tumors found in the breast specimen	
2	141 (77.0)
3^b^	27 (14.8)
4^c^	12 (6.6)
5^d^	3 (1.6)
No. of tumors assessed with IHC	
2	166 (90.7)
3	16 (8.7)
4	1 (0.5)
Size of the largest tumor, mm	
pT1	77 (42.1)
pT2	100 (54.6)
pT3	6 (3.3)
Nodal status	
pN0	106 (57.9)
pN1	40 (21.9)
pN2	10 (5.4)
pN3	5 (2.7)
pN_micro_	8 (4.4)
pN_ITC_	5 (2.7)
No axillary surgery performed	9 (4.9)
Adjuvant therapy received	
Endocrine therapy	168 (93.3)
Chemotherapy	64 (35.0)
Trastuzumab	21 (11.7)
Radiotherapy	93 (51.7)

Data are presented as number of specimens (%), except for age and adjuvant therapy received where data are presented as number of patients (%)

*IQR* interquartile range, *SLNB* Sentinel lymph node biopsy, *ALND* Axillary lymph node dissection, *IHC*  immunohistochemistry

^a^Sentinel lymph node biopsy was performed during primary surgery with additional axillary lymph node dissection at a later occasion

^b^Of the specimens with three primary tumors in the breast, 15 had immunohistochemistry (IHC) assessed on two tumors and 12 had IHC assessed on three tumors

^c^Of the specimens with four primary tumors in the breast, nine had IHC assessed on two tumors and three had IHC assessed on three tumors

^d^Of the specimens with five primary tumors in the breast, one had IHC assessed in two tumors, one had IHC assessed in three tumors, and one had IHC assessed in four tumors

### Characteristics of the tumor foci

The characteristics of PT1 and PT2 are shown in Table 2. Median size was 22.0 mm (IQR 14–30) for PT1 and 10.0 mm (IQR 7–15) for PT2. PT2 was the second largest focus in all specimens where two foci were assessed with IHC. For specimens where three or four foci were assessed with IHC, PT2 was the second largest tumor in all but two cases. In these two cases, PT2 was the focus with molecular surrogate subtype discordancy. The size of these two PT2s was only one and two mm smaller than the second largest. Both PT1 and PT2 were predominantly ductal (125 [68.3%] vs. 127 [69.4%]), Nottingham histologic grade 2 (111 [60.7%] vs. 106 [57.9%]), and luminal A (117 [63.9%] vs. 129 [70.5%]) for PT1 and PT2, respectively.

**Table 2:** Characteristics of the largest primary tumor (PT1) and second primary tumor (PT2) assessed with immunohistochemistry for patients with ipsilateral multifocal primary breast cancer

	PT1	PT2^1^
Tumor size, mm (median (IQR)	22.0 (14–30)	10.0 (7–15)
Pathologic tumor size		
pT1	77 (42.1)	153 (83.6)
pT2	100 (54.6)	30 (16.4)
pT3	6 (3.3)	0 (0.0)
Histological grade, NHG		
1	36 (19.7)	51 (27.9)
2	111 (60.7)	106 (57.9)
3	36 (19.7)	26 (14.2)
Histological type		
Ductal	125 (68.3)	127 (69.4)
Lobular	22 (12.0)	24 (13.1)
Tubular	15 (8.2)	20 (10.9)
Tubulolobular	7 (3.8)	7 (3.8)
Mixed ductal and lobular	12 (6.6)	3 (1.6)
Other	2 (1.1)	2 (1.1)
ER		
Positive	171 (93.4)	172 (94.0)
Negative	12 (6.6)	11 (6.0)
PR		
Positive	145 (80.9)	146 (79.8)
Negative	38 (19.1)	37 (20.2)
Ki67		
Low	129 (70.5)	146 (79.8)
High	54 (29.5)	37 (20.2)
HER2		
Positive	18 (9.8)	18 (9.8)
Negative	165 (90.2)	165 (90.2)
Subtype		
Luminal A	117 (63.9)	129 (70.5)
Luminal B HER2-	40 (21.9)	29 (15.8)
Luminal B HER2 +	14 (7.7)	14 (7.7)
Non-luminal HER2 +	4 (2.2)	4 (2.2)
Triple-negative	8 (4.4)	7 (3.8)

Data are presented as No. (%)

*ER* Estrogen receptor, *HER2* human epidermal growth factor 2, *IQR* interquartile range, *NHG* Nottingham histologic grade, *PR* progesterone receptor, *PT* primary tumor

^1^For the specimens where three or four foci were assessed with IHC, PT2 was the second largest tumor in all but two specimens. In these two specimens, the PT2 was the focus that had a molecular surrogate subtype discordancy compared to PT1. The sizes of these two PT2 tumors were only one and two mm smaller than the second largest tumor in the specimen, respectively

### Discordance in biomarker expression and molecular surrogate subtyping between PT1 and PT2

Discordance in ER, PR, HER2 and Ki67 was found in 2.7% (*n* = 5), 19.1% (*n* = 35), 7.7% (*n* = 14) and 16.9% (*n* = 31) of the 183 invasive foci, respectively. Ki67 was the only biomarker that showed significant changes in expression (*p* = 0.03) where 44.8% (24 of 54) changed from high expression in PT1 to low expression in PT2 (Table 3). The biomarker status was then used to determine the molecular surrogate subtypes, which were found to be concordant for 135/183 (73.8%) specimens and discordant for 48/183 (26.2%) specimens. The most common concordant molecular surrogate subtype between PT1 and PT2 was PT1 luminal A/ PT2 luminal A (102 of 135 [75.6.8%]), while the most common discordant molecular surrogate subtype was in the PT1 luminal B HER2-/PT2 luminal A group (21 of 48 [43.8.%]); Table 4).

**Table 3:**
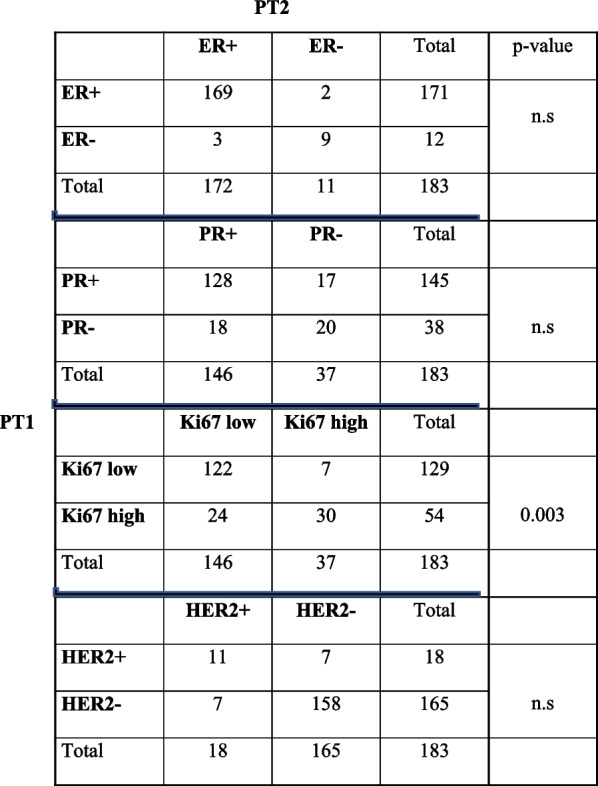
Concordance in breast cancer biomarkers between the largest tumor (PT1) and second tumor (PT2) assessed with immunohistochemistry for patients with multiple ipsilateral primary breast cancer

Data are presented as the number of specimens. *ER* Estrogen receptor, *HER2* Human epidermal growth factor 2, *PR* Progesterone receptor, *PT* Primary tumor; *p*-value based on McNemar’s test; n.s = not significant (*p*-value > 0.05)

**Table 4:** Concordance in molecular surrogate subtypes between the largest primary tumor (PT1) and the second tumor (PT2) assessed with immunohistochemistry in specimens with ipsilateral multifocal primary breast cancer

		PT2					
		Luminal A	Luminal B HER2-	Luminal B HER2 +	Non-luminal HER2 +	TNBC	Total
**PT1**	**Luminal A**	102	9	5	0	1	117
	**Luminal B HER2-**	21	17	2	0	0	40
	**Luminal B HER2 + **	4	2	7	1	0	14
	**Non-luminal HER2 + **	1	0	0	3	0	4
	**TNBC**	1	1	0	0	6	8
	**Total**	129	29	14	4	7	183

Data are presented as the number of specimens. *HER2* Human epidermal growth factor 2, *TNBC* Triple-negative breast cancer, *PT* Primary tumor

### Reevaluation of therapy decision making for cases with discordant molecular surrogate subtyping

**Fig. 2:**
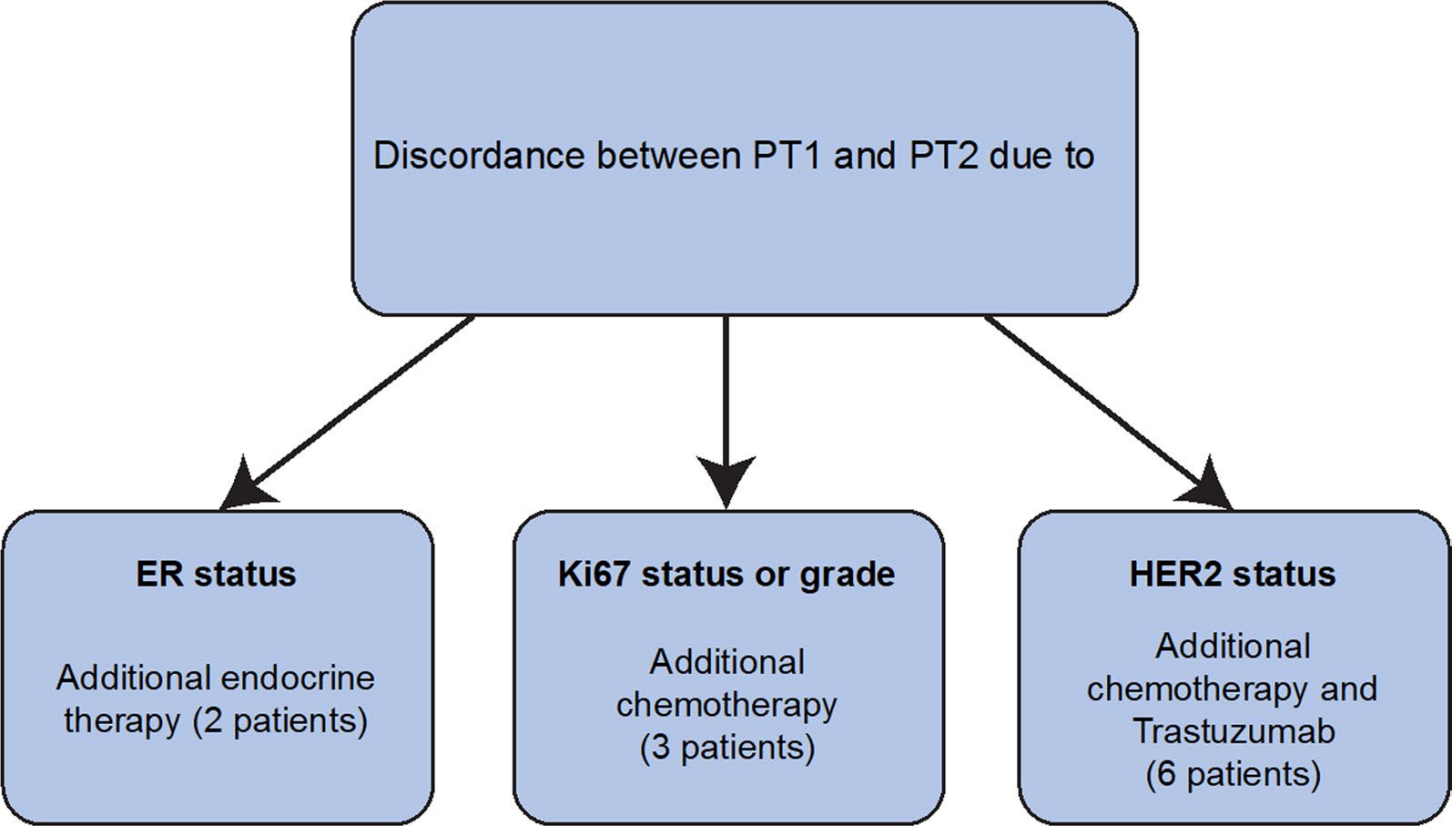
Clinical relevance of therapeutic changes due to discordance in molecular surrogate subtypes in the largest tumor (PT1) and the second tumor (PT2) in 11 of 180 patients with ipsilateral multifocal primary breast cancer that were surgically treated at Sahlgrenska University Hospital (Gothenburg, Sweden) between 2012 and 2017

**Fig. 3:**
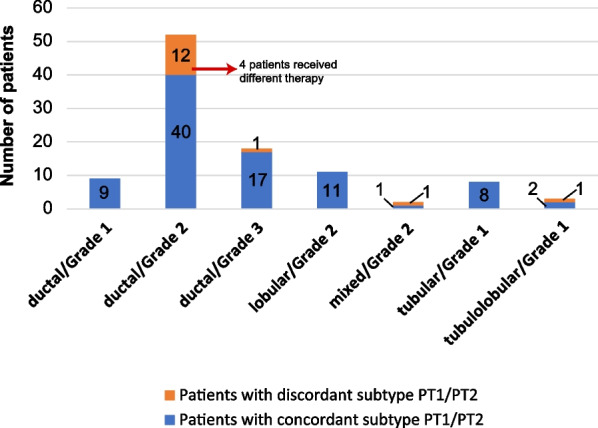
In total, 103 specimens with matching histologic type and grade (NHG) between the largest primary tumor (PT1) and second primary tumor (PT2) assessed with immunohistochemistry in specimens with ipsilateral multifocal primary breast cancer

The 48 discordant PT1 and PT2 specimens (48 patients) based on molecular surrogate subtyping were reevaluated for the previously recommended therapeutic options using a new multidisciplinary team board consisting of a medical oncologist (P.K), surgical oncologist (S.J) and a board-certified breast pathologist (A.K). Applicable guidelines at the time of surgery were considered. Eighteen patients (37.5%) had a more aggressive subtype in PT2 than PT1 of which PT1 luminal A/PT2 luminal B HER2- was the most common discordance (9 of 48 [18.6%]), and 30 (62.5%) had a less aggressive subtype in PT2 compared to PT1 (Table 5). Twenty patients (41.7% of the discordant 48 patients and 11.1% of the study cohort) had subtype changes in PT2, which potentially could have led to different therapy recommendations. Of the 20 patients with potential therapy changes, 11 (22.9% of the 48 discordant patients and 6.1% of the study cohort) were recommended therapy changes due to the subtype of PT2, i.e., two patients received additional endocrine therapy due to changes in ER status, three received additional chemotherapy due to changes in Ki67 status or tumor grade, and six received additional combined chemotherapy and trastuzumab due to HER2 positivity (Fig. 2). In addition, one patient would have received endocrine therapy, but due to the small tumor size of PT2 (5 mm) no treatment was recommended according to the Swedish national guidelines applicable for the current year. Another patient had a luminal B HER2 + PT2 but was not recommended trastuzumab due to the small tumor size (3 mm). In total, 37 patients (77.1% of the discordant patients and 20.1% of the study cohort) received no additional treatment due to either age, comorbidities, patient choice, or the patient received all possible treatment due to the status of PT1. Of the 11 patients where recommended therapy changed due to PT2, four patients had concordant histology and grade within the foci, and seven were discordant in histology and grade within the foci (Fig. 3).

**Table 5:** Discordance in subtypes between the largest primary tumor (PT1) and the second tumor (PT2) assessed with immunohistochemistry and patients where therapy was added due to the discordance in specimens with ipsilateral multifocal primary breast cancer

		PT2					
		Luminal A	Luminal B HER2-	Luminal B HER2 +	Non-luminal HER2 +	TNBC	Total
**PT1**	**Luminal A**	-	9 (2)^a,c^	5 (4)^a,c^	-	1 (1)^a,c^	15 (7)
	**Luminal B HER2-**	21^b^	-	2 (2)^a,c^	-	-	23 (2)
	**Luminal B HER2 + **	4^b^	2^b^	-	1^a^	-	7
	**Non-luminal HER2 +**	1^b^^,c^	-	-	-	-	1
	**TNBC**	1 (1)^b,c^	1 (1)^b,c^	-	-	-	2 (2)
	**Total**	27 (1)	12 (3)	7 (6)	1	1 (1)	48 (11)

Data are presented as number of patients with discordant subtypes (number of patients where therapy was added due to discordance in PT2)

*HER2* Human epidermal growth factor 2, *TNBC* Triple-negative breast cancer, *PT* Primary tumor

^a^more aggressive molecular surrogate subtype change in PT2 compared to PT1

^b^less aggressive molecular surrogate subtype change in PT2 compared to PT1

^c^patients with potential therapy changes

### Disease-free survival and overall survival

**Fig. 4:**
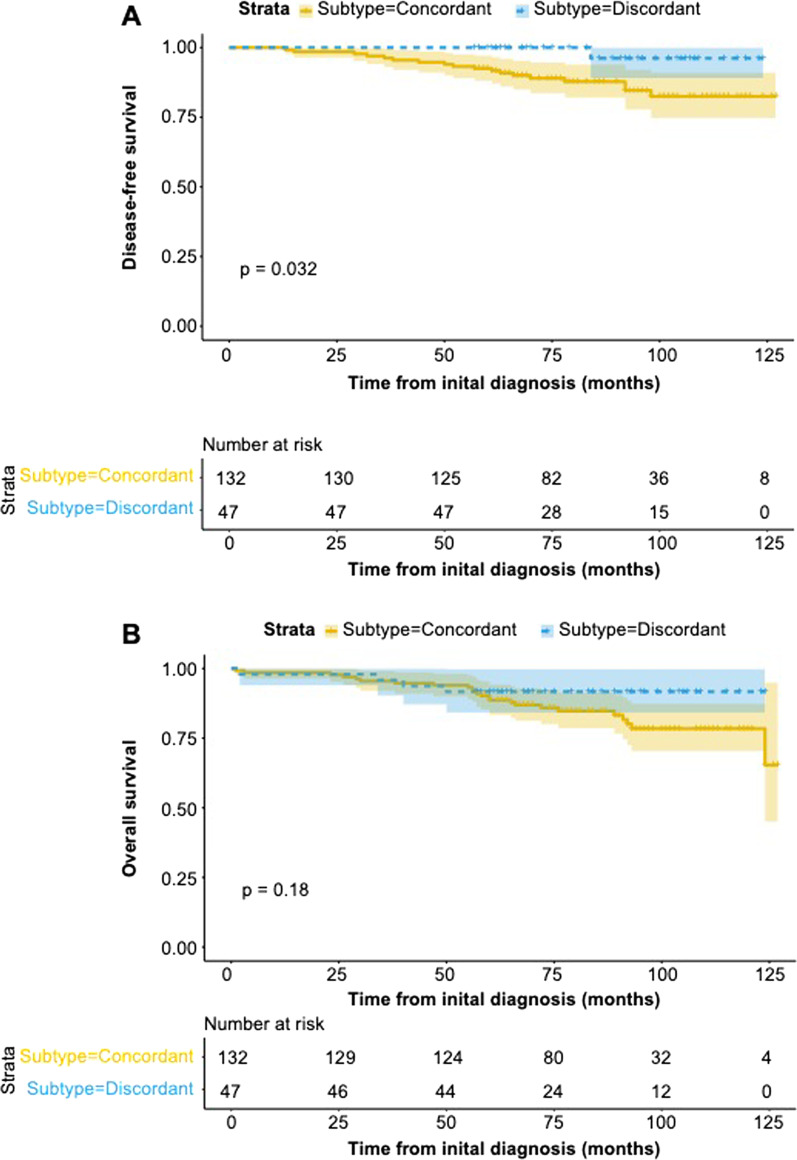
Comparison between concordant with discordant molecular surrogate subtypes for **A** disease-free survival (DFS) and **B** overall survival (OS) in patients with ipsilateral multifocal primary breast cancer (IMBC) surgically treated at Sahlgrenska University Hospital (Gothenburg, Sweden) between 2012 and 2017. One patient with IMBC was excluded from the analysis since the left-side specimen had concordant subtypes, while the right-side specimen had discordant subtypes between the different foci. This patient had no events

Survival analysis showed that the median follow-up time for the study cohort was 79.7 months (IQR 1.7–128.4). In addition, univariate analysis showed that the molecular surrogate subtype-concordant group had significantly worse DFS than the molecular surrogate subtype-discordant group (*p* = 0.032), but no significant difference in OS was found between these groups (*p* = 0.18) (Fig. 4A, B). Recurrence was observed in 19 patients, with 18 in the molecular surrogate subtype-concordant group (13.6%) and 1 (2.1%) in the molecular surrogate–discordant group (*p* = 0.027). Deaths were observed in 28 patients, of which 24 (18.2%) were in the molecular surrogate subtype-concordant group and 4 (8.5%) in the molecular surrogate discordant–group (*p* = 0.147).

### Specimens with matching histologic type and grade

In the study cohort, 103 specimens (103 patients) had the same histologic type and grade in the different foci. Yet, 15 (14.6%) of these patients had discordant molecular surrogate subtyping for PT1 and PT2. Notably, 4 of these 15 patients were recommended therapeutic changes due to PT2 (Fig. 3). We also conducted a comparison between the 103 patients with concordant morphology and grade, where ≥ 2 foci were assessed with IHC, to 102 excluded patients with similarly concordant features, but where only one focus was assessed with IHC (Additional file 1: Table 1). Compared to the group of 103 patients, the 102 excluded patients more frequently underwent axillary lymph node clearances (47.1% compared to 27.2%) and had a larger lymph node burden: 49.0% were pN0 compared to 55.3%. In addition, the 102 excluded patients more frequently had Grade 3 tumors (30.4% vs. 17.5%, *p* = 0.031), higher Ki67 levels (44.1% vs. 25.2%, *p* = 0.004) and received more chemotherapy (51.0% vs. 33.0, *p* = 0.009) than the group of 103 patients. Recurrence was observed in 10 (9.8%) patients in the excluded group of 102 patients and in 13 (12.6%) patients in the 103 patient group (*p* = 0.526). Deaths were recorded in 23 (22.5%) patients in the excluded group of 102 patients and in 21 (20.4%) patients in the 103 patient group (*p* = 0.715). Although the excluded group of 102 patients had more aggressive tumors, higher nodal burden and received more chemotherapy, univariate survival analysis showed no statistically significant difference in neither DFS (*p* = 0.56) nor OS (*p* = 0.62) when comparing these two groups with shared features considering histology and grade between the different foci (Additional File 2: Fig. 1A, B).

## Discussion

In this retrospective study, we found that 10.3% of the patients diagnosed and treated for primary breast cancer at Sahlgrenska University Hospital in Gothenburg, Sweden, between 2012 and 2017 had ipsilateral multifocal primary breast cancer, which is in line with a study by Buggi et al*.* [13] (9.3%). Although 308 specimens were eligible for inclusion in the study, only the 183 specimens (180 patients) that had IHC performed for at least two foci (PT1 and PT2) were included. Discordance in molecular surrogate subtype between PT1 and PT2 was found in 48 patients, and the additional multidisciplinary team board identified 20 (11.1%) patients that could potentially have had changed treatment recommendations due to the molecular surrogate subtype in PT2. However, in the clinical setting, 11 (6.1%) of the 20 patients actually received different/additional treatment.

Although we showed a subtype discordance of 26.2%, adjuvant treatment recommendations only changed for 11 patients due to PT2 subtype, four of the 11 patients had concordant histology and grade within the different foci, whereas seven had discordant histology and grade within the different foci. Two more patients had subtype changes that could have led to additional treatment but did not meet the criteria for receiving the actual treatment due to small tumor sizes. Thus, all subtype mismatches do not automatically lead to treatment changes. In our study, we could see that 37.5% of the foci with molecular surrogate subtype discordances had a more aggressive subtype in PT2, but the most common subtype difference was where PT2 was less aggressive compared to PT1 in molecular surrogate subtyping, primarily PT1 Luminal B HER2-/PT2 Luminal A. Nevertheless, 6.1% of the patients in our cohort were recommended additional treatment due to differences in subtype between the two foci. Buggi and colleagues found that 12.4% of patients received different adjuvant treatment due to foci heterogeneity. Our two studies are not completely comparable, since the study by Buggi and colleagues excluded multiple lesions with different histological features in the different foci [13].

There is no consensus whether it is necessary to assess all tumor foci found in a specimen with IHC. Mosbah et al*.* and Middleton et al*.* reported no differences in biomarker status [12, 29] between different foci, whereas other studies have shown differences [13, 25, 30]. Our results are in line with the latter studies and support routine evaluation of all foci with IHC. When the foci were classified into the molecular surrogate subtypes, we showed that 26.2% of the specimens had a subtype mismatch between PT1 and PT2. Choi et al. [30] showed a concordance in subtype for 92% of the 64 patients included in the study, while Li et al*.* [25] showed 84.9%. Both studies show higher concordance than the 73.8% in the present investigation. Even regarding the breast cancer biomarkers, Moshab et al. [29] and Middleton et al. [12] demonstrated no discordant cases for subtype. Therefore, larger studies should be conducted to determine the concordance of subtype between all foci in IMBC.

Although current guidelines do not support IHC assessment on all foci when they share the same morphology, our institution assessed approximately 50% of the specimens where same morphology was featured within the specimen during the study period (2012–2017). Despite shared histologic type and grade for PT1 and PT2 (*n* = 103), 15 specimens were discordant for molecular surrogate subtype and four patients would have been recommended additional adjuvant therapy. We recognize that there is a selection bias which prevents us from extrapolating these findings to the 125 excluded specimens where only one focus was assessed with IHC. Yet, a comparison between the 102 excluded patients with < 2 foci determined to have the same grade and histology, and 103 of the included patients with the same features, revealed no differences in OS or DFS, though high Ki67 levels, Grade 3, axillary lymph node dissection and chemotherapy were more prevalent in the 102 excluded specimens.

Patient survival is another topic associated with controversy for multifocal breast cancer. Most studies compare multifocal/multicentric breast cancer with unifocal breast cancer [5, 7, 15, 31]. Few studies have compared survival for cohorts with multiple tumors, i.e., subtype concordant versus subtype discordant samples. A study by Li et al*.* [25] only had a median follow-up time of 36 months, but showed that the concordant group generally had a better DFS and OS than the discordant group, though not statistically significant. In the present study, we analyzed DFS and OS for ipsilateral multifocal primary breast cancer patients with foci having discordant and concordant subtypes. Our median follow-up time was longer (80 months) and the survival curves showed the opposite trend, where the concordant group had both worse DFS as well as OS compared to the discordant group. However, multivariable analysis was not possible due to the low number of events. The most likely explanation is that there are imbalances between the patient and tumor characteristics, as well as the treatment received. Therefore, no conclusions can be drawn from these findings.

## Conclusion

We showed that subtype discordances occurred in 26.7% of patients with ipsilateral multifocal primary breast cancers, leading to changes in adjuvant treatment recommendations for 6.1% of the patients. These findings warrant further studies to assess the necessity of performing IHC on all tumor foci found in a breast cancer specimen, regardless of whether the different foci share the same histologic type and grade. This will ensure that patients with multifocal breast cancer are recommended appropriate individualized treatment.

## Supplementary Information

**Additional file 1**. **Supplementary Table 1**. Comparison between 103 (of the 183 included in the study) specimens with shared morphology and grade where ≥2 foci were assessed with IHC with the 102 excluded patients that also had the same morphology and grade but where only one focus was assessed with IHC. Data for size, histological type, histological grade, biomarkers and subtype refers to the largest tumor assessed with IHC for both groups

**Additional file 2**. **Supplementary Figure 1**. Comparison between 103 (of the 183 included in the study) specimens with shared morphology and grade where ≥2 foci were assessed with IHC with the 102 excluded patients that also had the same morphology and grade but where only one focus was assessed with IHC for **A **disease-free survival (DFS) and **B** overall survival (OS) in patients with ipsilateral multifocal primary breast cancer surgically treated at Sahlgrenska University Hospital (Gothenburg, Sweden) between 2012 and 2017

## Data Availability

The data supporting the findings of this study are available on reasonable request to the corresponding author.

## Abbreviations

DCIS: Ductal carcinoma in situ
ER: Estrogen receptor
HER2: Human epidermal growth factor 2
IHC: Immunohistochemistry
IMBC: Ipsilateral multifocal primary breast cancer
NHG: Nottingham histologic grade
PR: Progesterone receptor
SISH: Silver in situ hybridization
