# Supplementary material for: Clinical evaluation of molecular surrogate subtypes in patients with ipsilateral multifocal primary breast cancer
Source: Breast Cancer Res. 2023 Apr 6;25:36. doi: 10.1186/s13058-023-01632-5 (PMC10080895; doi:10.1186/s13058-023-01632-5)
Supplement: Supplementary file 1 — Additional file 1. Supplementary Table 1. Comparison between 103 (of the 183 included in the study) specimens with shared morphology and grade where ≥2 foci were assessed with IHC with the 102 excluded patients that also had the same morphology and grade but where only one focus was assessed with IHC. Data for size, histological type, histological grade, biomarkers and subtype refers to the largest tumor assessed with IHC for both groups [file 13058_2023_1632_MOESM1_ESM.docx]

**Supplementary Table 1.** Comparison between 103 (of the 183 included in the study) specimens with shared morphology and grade where >2 foci were assessed with IHC with the 102 excluded patients that also had the same morphology and grade but where only one focus was assessed with IHC. Data for size, histological type, histological grade, biomarkers and subtype refers to the largest tumor assessed with IHC for both groups.

|  | **1 focus assessed with IHC** | **≥2 foci assessed with IHC**^1^ | **p-value** |
| --- | --- | --- | --- |
| **No. of patients (n)** | 102 | 103 |  |
| **No. of specimens (n)** | 102 | 103 |  |
| **Age (median [IQR])** | 61.00 [48.00, 74.00] | 60.00 [52.00, 70.50] | 0.858 |
| **Surgery, breast (%)** |  |  | 0.169 |
| Breast conserving surgery | 40 (39.2) | 30 (29.1) |  |
| Mastectomy | 62 (60.8) | 73 (70.9) |  |
| **Surgery, axilla (%)** |  |  | 0.013 |
| SLNB | 53 (52.0) | 72 (70.0) |  |
| SLNB + ALND^2^ | 28 (27.5) | 14 (13.6) |  |
| ALND | 19 (18.6) | 14 (13.6) |  |
| No surgery performed | 2 (2.0) | 3 (2.9) |  |
| **Tumor size, mm (median [IQR])** | 24.00 [17.00, 40.75] | 24.00 [15.50, 32.00] | 0.145 |
| **Size of the largest tumor (%)** |  |  | 0.051 |
| pT1 | 35 (34.3) | 38 (36.9) |  |
| pT2 | 55 (53.9) | 62 (60.2) |  |
| pT3 | 12 (11.8) | 3 (2.9) |  |
| **Nodal status (%)** |  |  | 0.06 |
| pN0 | 50 (49.0) | 57 (55.3) |  |
| pN1 | 34 (33.3) | 26 (25.2) |  |
| pN2 | 11 (10.8) | 5 (4.9) |  |
| pN3 | 5 (4.9) | 4 (3.9) |  |
| pN_micro_ | 0 (0.0) | 5 (4.9) |  |
| pN _ITC_ | 0 (0.0) | 3 (2.9) |  |
| NA due to no axillary surgery performed | 2 (2.0) | 3 (2.9) |  |
| **Histological type (%)** |  |  | 0.213 |
| ductal | 71 (69.6) | 79 (76.7) |  |
| lobular | 22 (21.6) | 11 (10.7) |  |
| mixed (ductal and lobular) | 3 (2.9) | 2 (1.9) |  |
| tubular | 4 (3.9) | 8 (7.8) |  |
| tubulolobular | 2 (2.0) | 3 (2.9) |  |
| **Histological grade, NHG (%)** |  |  | 0.057 |
| Grade 1 | 12 (11.8) | 20 (19.4) |  |
| Grade 2 | 59 (57.8) | 65 (63.1) |  |
| Grade 3 | 31 (30.4) | 18 (17.5) |  |
| **ER (%)** |  |  | 0.249 |
| Positive | 88 (86.3) | 95 (92.2) |  |
| Negative | 14 (13.7) | 8 (7.8) |  |
| **PR (%)** |  |  | 0.839 |
| Positive | 77 (75.5) | 80 (77.7) |  |
| Negative | 25 (24.5) | 23 (22.3) |  |
| **HER2 (%)** |  |  | 0.813 |
| Positive | 14 (13.7) | 12 (11.7) |  |
| Negative | 88 (86.3) | 91 (88.3) |  |
| **Ki67 (%)** |  |  | 0.007 |
| Positive | 45 (44.1) | 26 (25.2) |  |
| Negative | 57 (55.9) | 77 (74.8) |  |
| **Subtype (%)** |  |  | 0.17 |
| Luminal A | 53 (52.0) | 69 (67.0) |  |
| Luminal B HER2- | 26 (25.5) | 16 (15.5) |  |
| Luminal B HER2+ | 9 (8.8) | 10 (9.7) |  |
| non-luminal HER2+ | 5 (4.9) | 2 (1.9) |  |
| TNBC | 9 (8.8) | 6 (5.8) |  |
| **Adjuvant therapy received (%)** |  |  |  |
| **Endocrine therapy** |  |  | 0.266 |
| Yes | 87 (85.3) | 94 (91.3) |  |
| No | 15 (14.7) | 9 (8.7) |  |
| **Chemotherapy** |  |  | 0.014 |
| Yes | 52 (51.0) | 34 (33.0) |  |
| No | 50 (49.0) | 69 (67.0) |  |
| **Trastuzumab** |  |  | 1 |
| Yes | 12 (11.8) | 12 (11.7) |  |
| No | 90 (88.2) | 91 (88.3) |  |
| **Radiotherapy** |  |  | 0.274 |
| Yes | 67 (65.7) | 59 (57.3) |  |
| No | 35 (34.3) | 44 (42.7) |  |

IQR = interquartile range; SLNB= Sentinel lymph node biopsy; ALND= Axillary lymph node dissection; IHC= immunohistochemistry; TNBC triple negative breast cancer

^1^ The data presented for tumor characteristics concerns the largest tumor (=PT1)

^2^ Sentinel lymph node biopsy was performed during primary surgery with additional axillary lymph node dissection at a later occasion
