# Supplementary material for: Clinical evaluation of molecular surrogate subtypes in patients with ipsilateral multifocal primary breast cancer
Source: Breast Cancer Res. 2023 Apr 6;25:36. doi: 10.1186/s13058-023-01632-5 (PMC10080895; doi:10.1186/s13058-023-01632-5)
Supplement: Supplementary file 2 — Additional file 2. Supplementary Figure 1. Comparison between 103 (of the 183 included in the study) specimens with shared morphology and grade where ≥2 foci were assessed with IHC with the 102 excluded patients that also had the same morphology and grade but where only one focus was assessed with IHC for A disease-free survival (DFS) and B overall survival (OS) in patients with ipsilateral multifocal primary breast cancer surgically treated at Sahlgrenska University Hospital (Gothenburg, Sweden) between 2012 and 2017 [file 13058_2023_1632_MOESM2_ESM.docx]

**
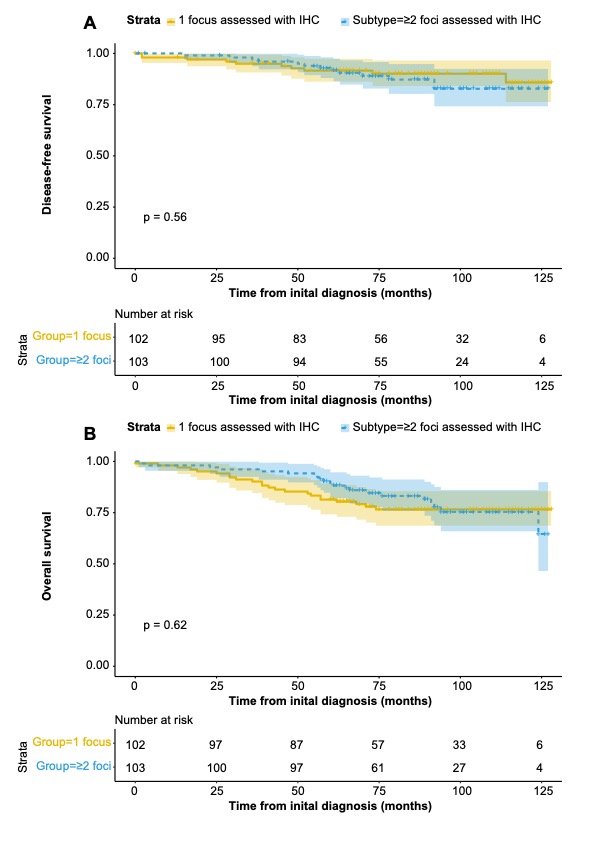
**

**Supplementary Figure 1.** Comparison between 103 (of the 183 included in the study) specimens with shared morphology and grade where >2 foci were assessed with IHC with the 102 excluded patients that also had the same morphology and grade but where only one focus was assessed with IHC for **A** disease-free survival (DFS) and **B** overall survival (OS) in patients with ipsilateral multifocal primary breast cancer surgically treated at Sahlgrenska University Hospital (Gothenburg, Sweden) between 2012 and 2017.
